# Cryo-EM Structures of Infectious Low-Twist α-Synuclein Filaments in Lewy Bodies

**DOI:** 10.64898/2026.09.16.752119

**Published:** 2026-09-17

**Authors:** Nicholas L. Yan, Mylan Mayer, Arthur A. Melo, Jacob I. Ayers, Daniel A. Mordes, William W. Seeley, Stanley B. Prusiner, Eric Tse, Vinita G. Chittoor-Vinod, Gregory E Merz

## Abstract

α-Synuclein (aSyn) aggregation within Lewy bodies underlies Parkinson's disease and associated dementias. These aggregates are often comprised of amyloid filaments, most of which appear untwisted or have extremely low twist, complicating high-resolution structural determination by cryogenic electron microscopy (cryo-EM). Here, we isolated filaments from the brain tissue of a patient with dementia with Lewy bodies and show infectivity in a cell line system that propagates Lewy aSyn. Then, we determined 2.3-2.9 Å cryo-EM structures of low-twist aSyn filaments from this tissue and from a publicly available Parkinson's disease dementia dataset. In both structures, the protofilament adopts a fold highly similar to the canonical Lewy fold with conserved cofactor and peptidic densities. Compared to previously resolved high-twist filaments, the low-twist filament is left-handed and flattened along the filament axis, resulting in register shifts of interlayer contacts, while preserving the overall architecture. Our results establish that low-twist filaments, which include over 75% of disease-associated Lewy aSyn amyloids, share a common structural core with high-twist forms and are the predominant aSyn species in samples that engage in templated infection.

## Full Text Availability

The license terms selected by the author(s) for this preprint version do not permit archiving in PMC. The full text is available from the preprint server.

